# In-hospital prescription changes and documentation in the medical records of the primary care provider: results from a medical record review study

**DOI:** 10.1186/s12913-017-2738-6

**Published:** 2017-11-29

**Authors:** Judith M. Poldervaart, Marije A. van Melle, Sanne Willemse, Niek J. de Wit, Dorien L.M. Zwart

**Affiliations:** 1 Julius Center for Health Sciences and Primary care, University Medical Center Utrecht, Utrecht University, Str. 6.101, PO box 85500, 3508AB Utrecht, the Netherlands; 2 University Medical Center Utrecht, Utrecht University, Utrecht, the Netherlands

**Keywords:** Prescription changes, Continuity of care, Transitional care, Patient safety, Primary care, Secondary care, Medical record

## Abstract

**Background:**

An increasing number of transitions due to substitution of care of more complex patients urges insight in and improvement of transitional medication safety. While lack of documentation of prescription changes and/or lack of information exchange between settings likely cause adverse drug events, frequency of occurrence of these causes is not clear. Therefore, we aimed at determining the frequency of in-hospital patients’ prescription changes that are not or incorrectly documented in their primary care provider’s (PCP) medical record.

**Methods:**

A medical record review study was performed in a database linking patients’ medical records of hospital and PCP. A random sample (*n* = 600) was drawn from all 1399 patients who were registered at a participating primary care practice as well as the gastroenterology or cardiology department in 2013 of the University Medical Center Utrecht, the Netherlands. Outcomes were the number of in-hospital prescription changes that was not or incorrectly documented in the medical record of the PCP, and timeliness of documentation.

**Results:**

Records of 390 patients included one or more primary-secondary care transitions; in total we identified 1511 transitions. During these transitions, 408 in-hospital prescription changes were made, of which 31% was not or incorrectly documented in the medical record of the PCP within the next 3 months. In case changes were documented, the median number of days between hospital visit and documentation was 3 (IQR 0–18).

**Conclusions:**

One third of in-hospital prescription changes was not or incorrectly documented in the PCP’s record, which likely puts patients at risk of adverse drug events after hospital visits. Such flawed reliability of a routine care process is unacceptable and warrants improvement and close monitoring.

## Background

Transitions between different healthcare settings put patients’ safety at risk. Transitions between primary and secondary care include referral, discharge after a hospital admission and outpatient clinic visits. The number of such patient transitions is likely to increase worldwide, caused by an aging patient population with increasing prevalence of multimorbidity receiving care at home, with an increasing number of professionals involved in transitional care pathways [1–3]. Indeed, there is a shift from secondary to primary care, triggered by a need to minimize healthcare costs without having to compromise on quality of care [4, 5].

Every care transition is a potential threat to continuity and safety, with potential risks, near misses and adverse events [6, 7]. Haggerty et al. divided the concept of continuity of care into three domains, namely managerial continuity, relational continuity and informational continuity [7]. Several studies found inadequate documentation and lack of communication between different care providers (thus informational (dis)continuity) to be common during care transitions, whilst timely transfer of the medical information after discharge is considered an essential condition for maintaining patient safety during health care transfers [8, 9].

Given the increase in polypharmacy in the last decade, one of the most challenging parts of exchanging patient information is to safeguard an accurate record of medication use of the patient at referral or discharge [10, 11]. Healthcare providers are often not fully updated with their patient’s actual medication use [12, 13]. For example, 27% of drugs that were stopped in the hospital because of adverse drug events were found to be re-prescribed after discharge by the primary care provider (PCP) [14]. Burnett et al. found a reliability of 81–87% for the several clinical systems studied (such as available outpatient information), of which 20% of reliability failures were associated with a potential risk of harm. [15] A gap in information is likely to cause medication errors, with the potential to cause patient harm and to result in additional healthcare costs [16]. Yet, knowledge on the frequency of breaches in the continuity of information on changes in patients’ medication prescriptions between primary and secondary care is scarce [17]. One reason for this is that until now in The Netherlands the electronic medical records (EMRs) of both settings mostly are not linked.

In the Netherlands, every citizen is listed with a PCP who serves as gatekeeper to the next levels of healthcare. The PCP, a trained family physician working a private general practice in the community, refers patients who need diagnostics or therapy that can solely be provided by medical specialists, to hospitals. Medical specialists see these patients in outpatient specialty care settings or after (acute) hospital admission. When a patient is discharged, or after visiting the outpatient specialty care, changes in treatment plans need to be adequately synchronized with the next care provider, −in the Netherlands always the PCP-, who provides concurrent care or follow-up and is generally the coordinator of care [18]. The in- and outpatient settings of the hospital work in same EMR, however, PCPs work independently from the hospital, cannot access this record and use different EMR systems. Transitional communication occurs mostly digitally, but also through paper mail and telephone.

So although in-hospital changes (during outpatient clinic visit, hospital admission or visit to the emergency department) should be communicated, letters are not always written or sent timely by the medical specialist, nor correctly received and processed by the PCP. Furthermore, patients may collect their new or changed prescriptions as prescribed by the medical specialist at the community pharmacist, but also at the hospital pharmacist. Only the community pharmacist is connected to the PCP’s record, and therefore medication prescriptions handled by the hospital pharmacist will not be transferred to the PCP record. These sub optimally connected local processes impede tracking the full medication journey from the in-hospital prescription change to actual registration and continuation in the primary care setting and thus impede assessing current performance of settings, as well as the possibility to measure effects of interventions.

Therefore, we aimed for determining the frequency of no or incorrect documentation of in-hospital patients’ prescription changes in their PCP’s medical record, by reviewing individual patient data in a research database with linked primary and secondary care EMRs.

## Methods

### Study design

A retrospective observational study was performed, using linked EMRs from one academic hospital and 40 of their primary care practices. This was a sub study of the Transitional Incident Prevention Program (TIPP) study. The TIPP study was designed to develop and test a program for enhancing patient safety during healthcare transitions between hospital and primary care. [19]

### Setting and patient selection

For this study we created a research database in which we linked patients’ primary and secondary care EMRs.

We conducted the current medical record study using data from 2013 at the cardiology and gastroenterology department of the University Medical Center Utrecht, a 1042-bed academic hospital, situated in Utrecht, a city in the Netherlands, and referring primary care practices affiliated with the Julius Huisartsen Network (Dutch network of PCPs participating in research). This linked record included the content of the EMR of both the PCPs (Promedico, Medicom) and hospital (Chipsoft); we used the contacts with the healthcare provider (i.e. free text fields), correspondence of the medical specialist to the PCP (e.g. discharge letters or outpatient clinic follow-up letters), and the prescription overviews. Inclusion criteria were adult patients who had transitioned from the hospital to their PCP or vice versa in 2013, e.g. who had been discharged from the two hospital departments (cardiology or gastroenterology) or had had contact with the hospital outpatient clinic or emergency room; and they were registered as a patient at one of our affiliated PCPs. Records were excluded when patients did not experience a care transition upon second look, or the medical record was empty. We randomly sampled 600 (43%) of the 1399 eligible patients for analyses. To secure data transfer in terms of privacy, selection, linking and pseudononymizing of the medical record data was all conducted by a trusted third party (ZorgTTP Houten, the Netherlands; a company specialized in secure linkage and pseudonimisation of data).

### Data collection

#### Transitions

Data was collected regarding age, sex and department that patients visited and the number of transitions. Transitions were categorized into a ‘hospital admissions’ group (at least 1 night in the hospital) and a ‘short hospital contacts’ group. The ‘short hospital contacts’ group consisted of three categories: (1) (telephone) contacts with the medical specialist of the outpatient clinic, (2) emergency room visits, or (3) short-stay hospital admissions (<1 day) in which medication was self-managed during the stay. Intercollegiate consultations were excluded as these did not involve patient transitions. If an outpatient clinic visit or emergency room visit was followed by a hospital admission, this visit was not counted separately, as then prescription changes would only be handed over to the PCP after discharge.

#### Prescription changes

Subsequently, every transition was separately assessed for changes in prescriptions. We defined changes as any alteration in medication during hospital admission or outpatient clinic visit, and divided these changes into the following 4 types also used by Uitvlugt et al.: prescription of new medication (‘Start’), termination of medication (‘Stop’), change in dose or frequency of a medication was changed (‘Change in frequency/dose’) or a change from one medication to another medication in the same medication group (‘Switch’) [17]. Changes in dermatological preparations, drugs prescribed for a short period (e.g. antibiotics) and over-the-counter medication were excluded, because these changes typically are more likely not to be communicated to the PCP, noted in the prescription overview of the PCP; knowing this makes interpretation difficult when these prescriptions are not noted. The type of changes, generic name, dosage and corresponding medication group was labelled according to the most commonly used medication database in the Netherlands [20].

#### Documentation of prescription change in record of PCP

Subsequently, we evaluated whether the in-hospital prescription changes were documented in the medical record of the PCP, either in the prescription overview or in the free text fields of the medical record. We had data available of every patient for the whole of 2013. Prescription changes should ideally be documented in the record of the PCP the next day after discharge, however, we checked up to three months after the change made in the record of the medical specialist. For each prescription change we calculated the number of days between discharge or outpatient clinic visit and entry in the medical record of the PCP. If no (correct) documentation of the prescription change was found, possible explanations (such as a temporarily change) were explored by further studying the linked medical records.

### Outcomes

The primary outcome was the percentage of in-hospital prescription changes that was not or incorrectly documented in the medical record of the PCP. We distinguished three different types of documentation regarding prescription changes. First, when a prescription change (in terms of name, dosage and frequency) was present and correctly noted in the prescription overview of the PCP and also correctly noted in the free text fields, this was classified as “Correct documentation”. Second, an absent or incorrect notation in the prescription overview, but with a correct notation in the free text field was classified as “Inadequate documentation”, as well as correct notation in the prescription overview but incorrect notation in the free text fields: it is better than no documentation at all, but still concerns a discontinuity in information or even incorrect information. Since in daily practice the prescription overview and free text fields both are used by the practice nurse or primary care provider, this presents a potential risk for patient safety. Last, an absent or incorrect notation in both the prescription overview and free text fields was classified as “No documentation”; the latter type corresponded to our primary outcome. An example of this primary outcome was when a prescription was stopped by a cardiologist, but still prescribed later on by the PCP.

Secondary outcome was the time lag between prescription change and documentation in the medical record of the PCP, which for safety reasons ideally would be implemented in the prescription overview of the PCP within one day. We calculated the median number of days and benchmarked the time lag against the cut-off point of one day, two weeks (the duration for which new medications normally are prescribed at the pharmacy in The Netherlands) and three months (the maximum duration for which medications are prescribed). Subsequently, the number and characteristics of transitions, prescription changes and medication groups involved in prescription changes were assessed, as well as the age and sex of patients.

### Statistical analysis

Continuous variables are presented as means (± standard deviation, SD) or medians (interquartile ranges, IQR) when data was not normally distributed, while categorical variables are presented as numbers (percentage). All data were analyzed using IBM SPSS Statistics version 21.

## Results

### Patient characteristics, transitions and prescription changes

**Fig. 1:**
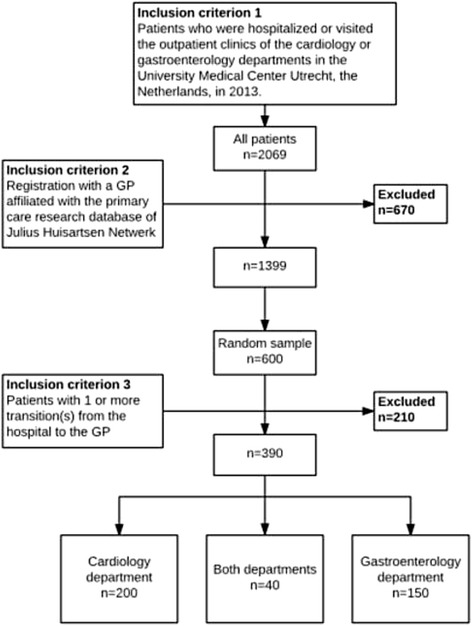
Flowchart of selected patients for assessment of reliability of the clinical system of in-hospital prescription changes and the documentation in the medical record of the PCP. PCP*: primary care provider; Julius Huisartsen Netwerk: Dutch network of* PCP*s participating in research*

In total, 2069 patients were hospitalized or had visited the outpatient clinics of the cardiology and gastroenterology department in 2013 (Table 1, Fig. 1). Of those, 670 patients were not registered as a patient at one of our affiliated PCPs, thus 1399 patients remained in our linked database, of which a random sample of 600 was extracted. Of those, 210 patients did not have a care transition upon second look, or the medical record was empty, and were excluded. Thus, 390 patients remained eligible for final analysis, of which 54% was male and the mean age was 59 years (SD 17). Of all patients, 200 (51%) visited the cardiology department, 150 patients (39%) the gastroenterology department and 40 patients (10%) visited both departments. In the records of these 390 patients we identified a total number of 1511 transitions from the hospital to the PCP; 68 (5%) of these were hospital admissions and 1443 (95%) were short hospital visits. We identified 408 prescription changes in 282 (19%) of these transitions, more often after admissions than after short hospital visits (Table 1).

**Table 1:** Patient and transition characteristics

Patients		390
Mean age, years (SD)		59 (±17)
Age ≤ 45		68 (16.7%)
Age 46–64		144 (35.3%)
Age ≥ 65		196 (48.0%)
Male		212 (54.4%)
Department		
Cardiology		200 (51.3%)
Gastroenterology		150 (38.5%)
Patients treated at both the cardiology and gastroenterology department		40 (10.2%)
Hospital admissions	Number of transitions	68
	*With prescription changes*	45 (66.2%)
	Department	
	Cardiology	42 (61.8%)
	Gastroenterology	26 (38.2%)
	Mean duration of stay in days (SD)	4.4 (±4.6)
Short hospital contacts^a^	Number of transitions	1443
	*With prescription changes*	237 (16.4%)
	Department	
	Cardiology	750 (52.0%)
	Gastroenterology	693 (48.0%)
Total	Number of transitions	1511
	*With prescription changes*	282 (18.7%)
	Department	
	Cardiology	792 (52.4%)
	Gastroenterology	719 (47.6%)

*SD* standard deviation

^a^Outpatient clinic contacts, emergency room visits and short-stay hospital admissions

### Documentation of prescription changes

In 126 (31%) of the 408 prescription changes, the change was not or incorrectly documented in the PCP’s medical record (“No documentation”) (Table 2). For 27 (21%) of the 126 prescription changes, a possible explanation could be found in the medical record. A description of these explanations are displayed in Appendix 1.

**Table 2:** Type of documentation of prescription changes, divided by the type of prescription change (start, stop, dose/frequency change and switch)

	Correct documentation Correct notation in prescription overview of PCP Correct notation in free text fields	Inadequate documentation Incorrect/no notation in prescription overview of PCP; Correct notation in free text fields OR Correct notation in prescription overview of PCP; Incorrect notation in free text fields	No documentation Incorrect/no notation in prescription overview of PCP Incorrect/no notation in free text fields
Total number of changes: *n* = 408 (100%)	86 (21.1%)	196 (48.0%)	126 (30.9%)
Type of change within the types of documentation			
Start^a^ *n* = 220 (53.9%)	43/86 (50.0%)^e^	117/196 (59.7%)	60/126 (47.6%)
Stop^b^ *n* = 77 (18.9%)	21/86 (24.4%)	43/196 (22.0%)	13/126 (10.3%)
Dose/freq change^c^ *n* = 87 (21.3%)	17/86 (19.8%)	24/196 (12.2%)	46/126 (36.5%)
Switch^d^ *n* = 24 (5.9%)	5/86 (5.8%)	12/196 (6.1%)	7/126 (5.6%)

*PCP* primary care provider

^a^A new medication was added

^b^A medication was stopped

^c^The dose or frequency of a medication was changed

^d^There was a switch from one medication to another in the same medication group

^e^Percentages shown are the type of change (e.g. “Start”) divided by the total number of changes in this particular type of documentation (e.g. “Correct documentation”)

In 86 (21%) of the 408 prescription changes, this was noted correctly in the prescription overview of the medical record (“Correct documentation”). In 196 (48%) changes, the change was not or incorrectly noted in the prescription overview of the PCP but was mentioned in free text fields (“Inadequate documentation”). Of those 408 changes, the type of change concerned the start of medication in 220 (54%), in 77 (19%) the stopping of medication, in 87 (21%) the change of dose or frequency of prescription, and in 24 (6%) it concerned switch of medication.

In the incorrectly documented prescription changes, the type of change mostly concerned the start of prescription (*n* = 60, 48%) or the change of dose or frequency of prescription (*n* = 46, 37%).

### Documentation specified for different patient groups

Documentation of prescription changes differed between age groups: among patients younger than 45 the prescription change was not documented in 40%, and for patients of 65 or older in 24% (Table 3). Documentation also differed between departments: no documentation occurred for 45% of the patients visiting the gastroenterologist, against 25% visiting the cardiologist.

**Table 3:** Documentation of prescription changes in the record of the primary care provider, for different patient groups

	Total number of prescription changes	Correct documentation	Inadequate documentation	No documentation
Sex				
Women	*n* = 187	38 (20.3%)	93 (49.7%)	56 (30.0%)
Men	*n* = 221	48 (21.7%)	103 (46.6%)	70 (31.7%)
Age				
≤ 45	*n* = 68	5 (7.4%)	36 (52.9%)	27 (39.7%)
46–64	*n* = 144	30 (20.8%)	62 (43.1%)	52 (36.1%)
≥ 65	*n* = 196	51 (26.0%)	98 (50.0%)	47 (24.0%)
Department				
Cardiology	*n* = 289	69 (23.9%)	148 (51.2%)	72 (24.9%)
Gastroenterology	*n* = 119	17 (14.3%)	48 (40.3%)	54 (45.4%)

### Timeliness of documentation

If changes were adequately documented in the record of the PCP, this happened within a median number of 3 days (IQR 0–18) after hospital visit or discharge (Table 4). For 26% of the patients, the change was documented within a day, for 41% within two weeks and for 55% within three months.

**Table 4:** Timeliness of documentation of prescription changes in the record of the primary care provider

Documentation		Start^a^ *n* = 220	Stop^b^ *n* = 77	Dose/freq change^c^ *n* = 87	Switch^d^ *n* = 24	Total *n* = 408/331^f^
Prescription overview	Number of days, median (IQR)^e^	2 (0–16)	n/a^g^	5 (0–33)	2 (1–14)	3 (0–18)
	≤ 1 day	64 (29.1%)	n/a	15 (17.2%)	6 (25.0%)	85 (25.7%)
	≤ 2 weeks	99 (45.0%)	n/a	24 (27.6%)	12 (50.0%)	135 (40.8%)
	≤ 3 months	129 (58.6%)	n/a	37 (42.5%)	16 (66.7%)	183 (55.3%)
Free text fields	Number of days, median (IQR)^e^	7 (4–14)	3.0 (1–9)	4 (1–24)	2 (1–8)	5 (2–14)

^a^A new medication was added

^b^A medication was stopped

^c^The dose or frequency of a medication was changed

^d^There was a switch from one medication to another in the same medication group

^e^Median number of days before documentation of the prescription change. Undocumented prescription changes are not included in this analysis

^f^This number is the total number of prescription changes. Second number is the number of prescription changes that should have been documented (minus ‘stop’). Only the prescription changes that should have been documented are used for calculating percentages

^g^In this category, timeliness could not be assessed, since when a prescription is stopped, this can only be assessed in the PCP’s record after 3 months,, when we could see in the prescription overview of the PCP that the patient did not receive the stopped prescription as a recurrent prescription (since the PCP had stopped the prescription)

We observed small differences between the type of change and median number of days of documentation, namely 2 for start and switch versus 5 for dose or frequency change. We observed the same trend for the timeliness of 1 day, 2 weeks and 3 months, with the dose and frequency change being less often documented within those cut-offs. For example, when a prescription change was started or switched, 59% and 67% was registered in the prescription overview of the PCP within 3 months, compared to 43% for dose or frequency change (Table 4).

### Medication groups

**Fig. 2:**
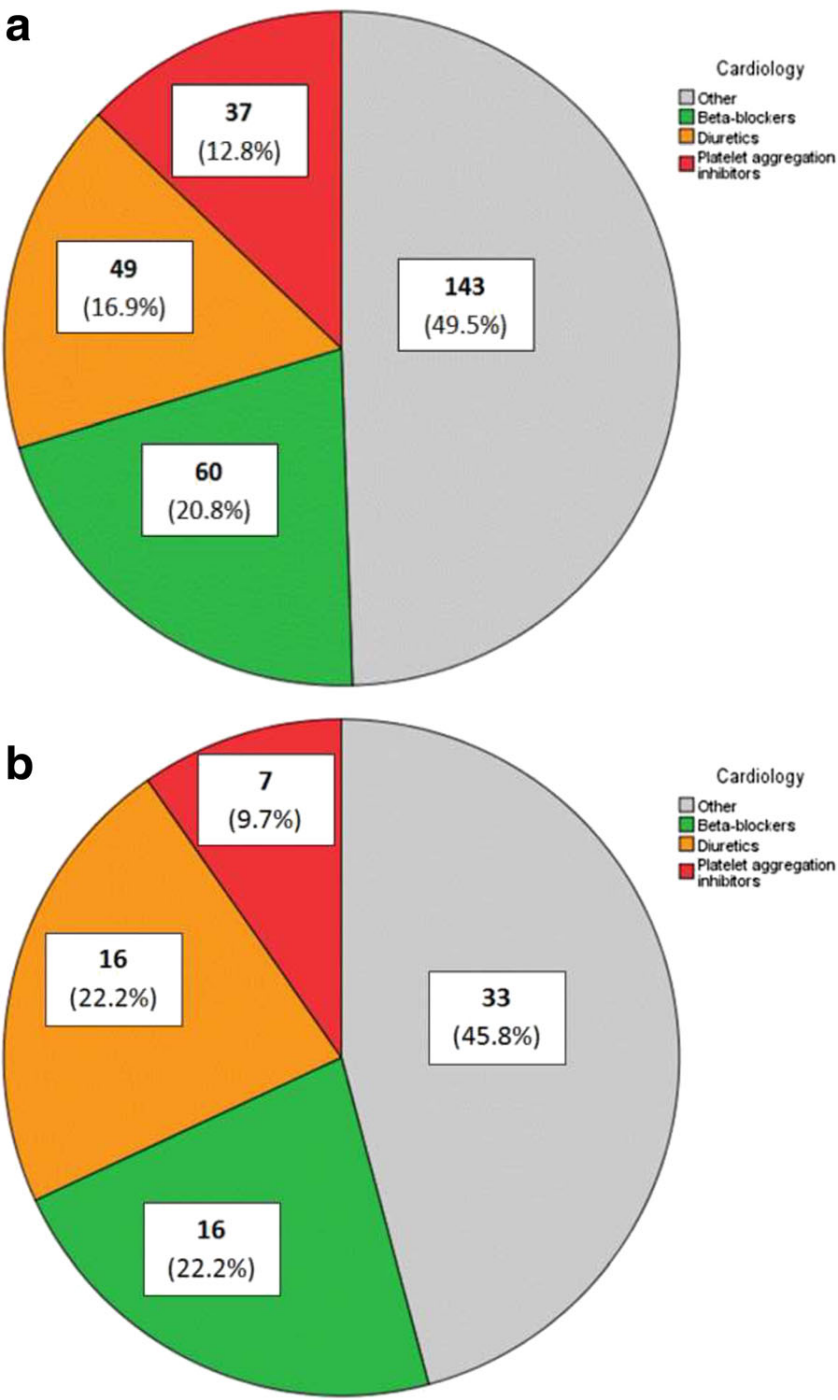
**a** Distribution of total prescription changes per medication group for patients treated at the cardiology department (*N* = 289). **b** Distribution not or incorrectly documented prescription changes per medication group for patients treated at the cardiology department (*N* = 72)

**Fig. 3:**
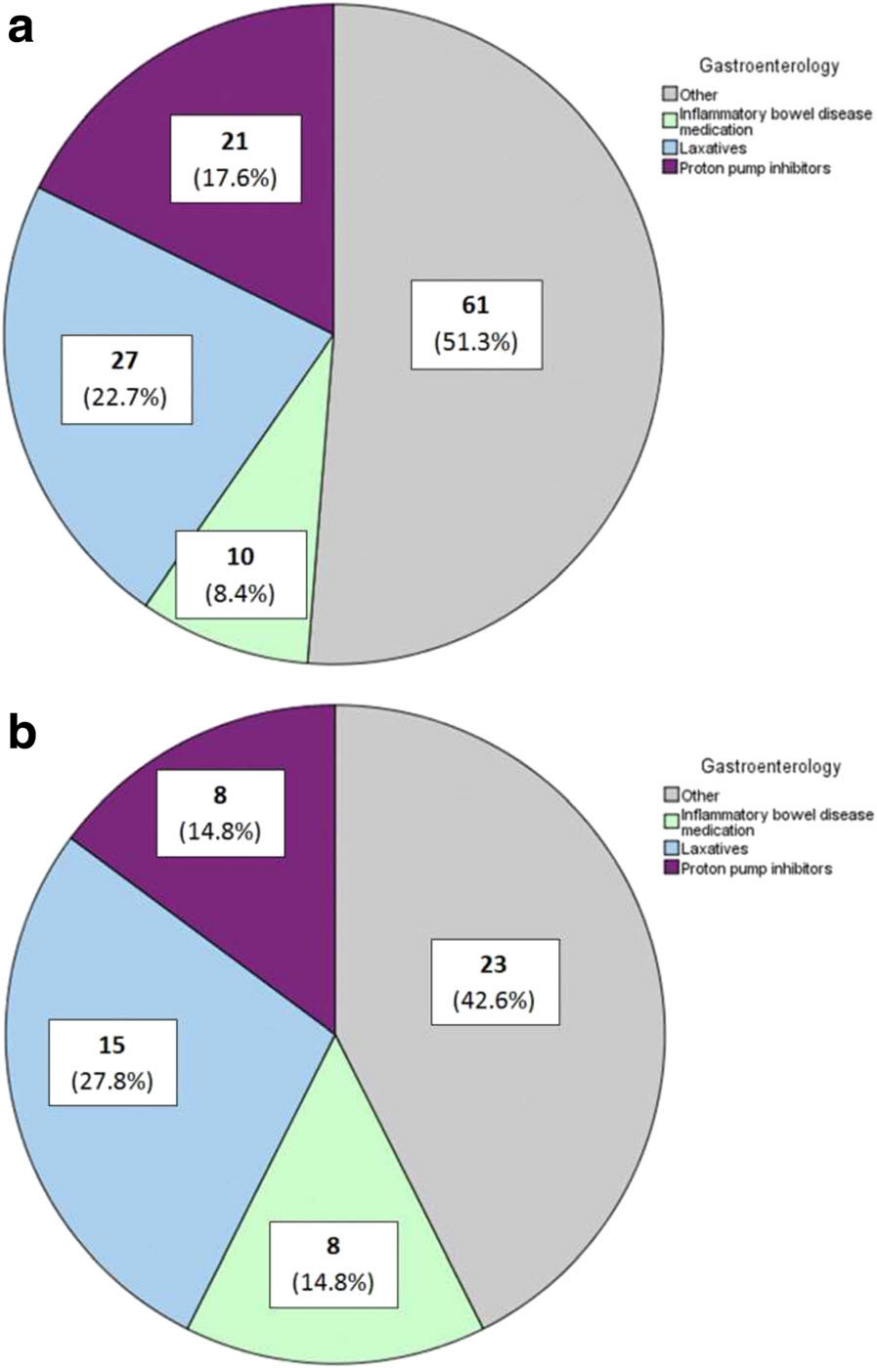
**a** Distribution of total prescription changes per medication group for patients treated at the gastroenterology department (*N* = 119). **b** Distribution of not or incorrectly documented prescription changes per medication group for patients treated at the gastroenterology department (*N* = 54)

For the cardiology department, 23 different groups of medication were involved in changes, of which three groups of medication were responsible for half of all prescription changes (Fig. 2a). These consisted of beta-blockers (21%), diuretics (17%) and platelet aggregation inhibitors (13%). For the gastroenterology department, 19 different groups of medication in total were involved, of which laxatives (23%), proton pump inhibitors (18%) and medication for the treatment of inflammatory bowel disease (8%) were the three medications mostly changed (Fig. 3a, Table 5).

**Table 5:** Medication groups involved in prescription changes and the percentage of no documentation in the record of the primary care provider

Department	Medication groups involved in prescription changes N = 408		No documentation Incorrect/no notation in prescription overview of PCP Incorrect/no notation in free text fields *N* = 126
Cardiology	Total	N = 289	N = 72
	1. Beta-blockers	60 (20.8%)	16 (22.2%)
	2. Diuretics	49 (16.9%)	16 (22.2%)
	3. Platelet aggregation inhibitors	37 (12.8%)	7 (9.7%)
	Other	143 (49.5%)	33 (45.8%)
Gastroenterology	Total	N = 119	N = 54
	1. Laxatives	27 (22.7%)	15 (27.8%)
	2. Proton pump inhibitors	21 (17.6%)	8 (14.8%)
	3. Inflammatory bowel disease medication	10 (8.4%)	8 (14.8%)
	Other	61 (51.3%)	23 (42.6%)

*PCP* primary care provider

Comparing the medication groups involved in total changes versus the medication groups involved in changes that were not documented, medication relatively more often not documented were beta-blockers and diuretics (44% of not documented prescription changes by the cardiologist, Fig. 2b) and laxatives (28% of not documented prescription changes by the gastroenterologist (Fig. 3b, Table 5).

## Discussion

We reviewed the transitional documentation of prescription changes in patients’ medical records, and found that one third of all in-hospital prescription changes were not documented in patient’s medical record of the PCP. Of these, in 80% of cases we could not trace a satisfying underlying reason (e.g. temporarily given medication) in the medical record of the medical specialist. Changes mostly concerned the start of a prescription in 53.9% (total changes) and 47.6% (not documented changes). When prescription changes were documented correctly, they were found after a median of three days (IQR 0–18.3) in the PCP’s patient records. The types start and switch were more often documented timely than the type dose or frequency change.

Our current findings are grossly in line with other studies. Another Dutch study showed a breakdown in information in each step of the discharge communication process, with only 50% of all prescription changes correctly documented in PCPs’ overviews two weeks after discharge. [17] This higher rate of incorrect documentation might reflect the shorter follow-up of two weeks, the use of medication reconciliation with the patient (including over-the-counter medication), and the fact that only discharge was assessed (and not also the outpatient clinic transition).

Despite Dutch multidisciplinary guidelines on information transfer from hospital to PCP and on transfer of medication, the criterion of informing the next healthcare provider within 24 h was not fulfilled [21, 22]. Two small surveys showed that 25% of the PCPs did not receive any communication from the hospital within 4 weeks after visit to the outpatient clinic [23, 24]. Bell et al. found that for 58% of all discharge summaries the goal of sending the letters within two weeks was not reached [25]. Kripalani et al. reported that 51–77% of the discharge summaries is not received by the PCP after four weeks, affecting the quality of care in 25% of follow-up visits [26]. However, this research concerns the deliverance of information from the hospital, and not the subsequent registration of this information in the PCP’s medical record, as assessed by us.

Problems with (timely) deliverance of sufficient information on the part of the hospital are partially responsible for the found high level of undocumented prescription changes. Undoubtedly it is not the only weak link in the process. The ability of the PCP to correctly process prescription changes to the records may be influenced by the amount of time available for administrative tasks, the information processing system and the level of collaboration with the community pharmacist [27, 28]. We found a relatively high number of documentation of prescription changes within one day. This is most likely due to automated documentation by the pharmacist’s system that currently in the Netherlands often is connected with the PCP’s. A comprehensive and reliable system implementing all prescription changes directly into the medical record of the PCP and/or pharmacist would be a definite solution.

Notably, we found unexpected differences between prescription changes made by the gastroenterologist and the cardiologist, as well as the fact that younger patients seem to be more at risk for absent documentation. A possible explanation may be that many older patients have automated dispensing systems (Baxter), providing the patient with the correct medications on each moment of drug intake during the day, and which are closely monitored by ‘own’ pharmacists. In contrast, younger patients could pick up their medication anywhere without communication to the PCP or their regular pharmacist. However, this finding should be interpreted cautiously, as statistically testing and correcting for interactions was not feasible in our sample.

Our study has several strengths. In contrast to other record studies, we studied the documentation of prescription changes in linked primary-secondary care medical records. Combining patients’ primary and secondary care EMRs provided us with a more complete picture of the implementation of prescription changes on the receiving side. Furthermore, we looked at all care transitions; not only hospital discharge but also outpatient clinic visits, emergency room visits and short stay hospital admissions. Third, we looked at the prescription overview in the medical record as well as free text fields of the medical record of the PCP, providing us optimal information of the medication. Finally, when documentation was absent or incorrect, we tried to find an explanation in the medical record, improving clinical accuracy of interpretation of our results.

Yet, our study also has certain limitations. First, since this concerns a retrospective study, we could often not establish the intention of a prescription change by the medical specialist in the hospital’s medical record. However, this does still not diminish the found results of undocumented prescription changes. Second, adverse drug events such as allergies and serious side effects were not included in our outcome, since data was not available to assess this. This would have made our outcome even more clinically relevant. Third, certain patient characteristics (e.g. educational level or presence of comorbidity) were unavailable, which limited our options to explore which patients were more vulnerable for insufficient transfer of information. Last, this concerned a single center study, decreasing generalizability.

When classifying documentation as in this study, it is important to keep in mind that insufficient quality of documentation is not necessarily insufficient quality of care. An individual case in which a prescription change was made due to an important clinical reason (e.g. a drug allergy) is handled equally as a prescription change that has a lower level of clinical importance (e.g. start of a laxative). Also, the pharmacist or the PCP often intercepts deficits in documentation before and corrects the missing information ‘outside the registration’ before any harm can be done to the patient. Results should therefore not be interpreted as a direct reflection of medical errors or poor quality of care, but as a reflection of documentation quality.

Various literature reviews demonstrate that discharge information is often insufficient or incomplete. A recent review including 19 studies (2007–2014) showed that 21% of the discharge summaries did not include post-discharge medication [29]. This might partly explain why these changes do not end up in the PCP’s medical record. Moreover, doctors learn to deal with poor documentation on a daily basis and are well capable of preventing patient harm caused by those documentation flaws in most cases. This level of resilience probably is the main explanation as to why outcomes on documentation differ from outcomes on medical errors and adverse events [30]. However, we do hypothesize that this informational discontinuity will have its effect on patient safety. The more flaws in the healthcare process occur, the higher the risk for fatal slips [31]. Undocumented prescription changes unnecessarily increase the risk in the patient journey. Unique in this study is the use of a linked EMR, providing a more complete view on this patient journey between different healthcare settings, which was used to measure the extent of the problem, as well as trying to find reasons for undocumented prescription changes. We did provide insight in the extent of the problem, even though causes for omission were mostly not found. Both outcomes are a start for improvement strategies, in order to decrease the risk for harm for patients. The ultimate solution, which would be a single shared EMR through all levels of care, is up until now not within sight in the Netherlands.

## Conclusions

To conclude, one third of the prescription changes after hospital visits was not found in the medical record of the PCP. Additionally, when changes were correctly documented it was often not within the debatable timeframe of two weeks. It is likely that failures in adequate transfer of prescription changes do unnecessarily put the patients’ health at risk. Understanding the background for incorrect or absent documentation of prescription changes can guide us towards designing interventions that sufficiently address these patient safety issues.

## Appendix

**Table 6:** Explanations for incorrect or no documentation in the medical record of the primary care provider

No documentation Incorrect/no notation in prescription overview of primary care provider Incorrect/no notation in free text fields	126 (100%)
Possible explanation	27 (21.4%)
Change was temporarily made (e.g. antibiotics)	10
Change made within two weeks before end 2013 (data not available for 2014)	4
Acetylsalicylic acid prescribed instead of carbasalate calcium	3
Patient went to a different care provider shortly after discharge	3
Change was reversed by patient	3
Change regarded the number of days a fentanyl patch could be worn	3
Dosage was changed but was already prescribed in ‘new’ dosage (so primary care provider already had new dosage in medical record)	1
No explanation	99 (78.6%)

## Abbreviations

EMR: electronic medical record
IRQ: interquartile ranges
PCP: primary care provider

## References

[1] Bodenheimer T (2008). Coordinating care: a perilous journey through the health care system. N Engl J Med.

[2] Koné Pefoyo AJ, Bronskill SE, Gruneir A, Calzavara A, Thavorn K, Petrosyan Y (2015). The increasing burden and complexity of multimorbidity. BMC Public Health.

[3] Barnett K, Mercer SW, Norbury M, Watt G, Wyke S, Guthrie B (2012). Epidemiology of multimorbidity and implications for health care, research, and medical education: a cross-sectional study. Lancet.

[4] Vos L, Chalmers SE, Duckers ML, Groenewegen PP, Wagner C, van Merode GG. Towards an organisation-wide process-oriented organisation of care: a literature review. Implement Sci. 2011; 6.8–5908–6-8 10.1186/1748-5908-6-8 PMC3035025 21247491

[5] Starfield B, Shi L, Macinko J (2005). Contribution of primary care to health systems and health. Milbank Q.

[6] M Naylor, SA. Keating. Transitional care: moving patients from one care setting to another. Am J Nurs 2008; 108(9 Suppl): 58–63. 10.1097/01.NAJ.0000336420.34946.3a PMC2768550 18797231

[7] Join commission. Transitions of Care: The need for a more effective approach to continuing patient care… Hot Topics in Health Care. June 27, 2012. https://www.jointcommission.org/hot_topics_toc/

[8] Jones CD, MB V, O'Donnell CM, Anderson ME, Patel S, Wald HL (2015). A failure to communicate: a qualitative exploration of care coordination between hospitalists and primary care providers around patient hospitalizations. J Gen Intern Med.

[9] Göbel B, Zwart D, Hesselink G, Pijnenborg L, Barach P, Kalkman C (2012). Stakeholder perspectives on handovers between hospital staff and general practitioners: an evaluation through the microsystems lens. BMJ Qual Saf.

[10] Fulton MM, Riley AE (2005). Polypharmacy in the elderly: a literature review. J Am Acad Nurse Practitioners.

[11] Lemmens LC, Weda M. Polyfarmacie bij kwetsbare ouderen: risico’s rondom overgangen tussen eerste- en tweedelijnszorg (in Dutch). RIVM. 2015;

[12] Coleman EA, Smith JD, Frank JC, Eilertsen TB, Thiare JN, Kramer AM (2002). Development and testing of a measure designed to assess the quality of care transitions. Int J Integr Care.

[13] Cornish PL, Knowles SR, Marchesano R, Tam V, Shadowitz S, Juurlink DN (2005). Unintended medication discrepancies at the time of hospital admission. Arch Intern Med.

[14] Van der Linden CM, Kerskes MC, Bijl AM, Maas HA, Egberts AC, Jansen PA (2006). Represcription after adverse drug reaction in the elderly: a descriptive study. Arch Intern Med.

[15] Burnett S, Franklin BD, Moorthy K, Cooke MW, Vincent C (2012). How reliable are clinical systems in the UK NHS? A study of seven NHS organisations. BMJ Quality Safety.

[16] van der Stelt CA, Vermeulen Windsant-van den Tweel AM, Egberts AC, van den Bemt PM, Leendertse AJ, Hermens WA, et al. The association between potentially inappropriate prescribing and medication-related hospital admissions in older patients: a nested case control study. Drug Saf 2016;39(1):79–87. 10.1007/s40264-015-0361-1 26553305

[17] Uitvlugt EB, Siegert CEH, Janssen MJA, Nijpels G (2015). Karapinar-Carkit. Completeness of medication-related information in discharge letters and post-discharge general practitioner overviews. Int J Clin Pharm.

[18] Cresswell KM, Sadler S, Rodgers S, Avery A, Cantrill J, Murray SA, et al. An embedded longitudinal multi-faceted qualitative evaluation of a complex cluster randomized controlled trial aiming to reduce clinically important errors in medicines management in general practice. Trials 2012;13.78–6215–13-78. 10.1186/1745-6215-13-78 PMC3503703 22682095

[19] Van Melle MA, Zwart DLM, de Bont A, Mol IWM, van Stel HF, de Wit NJ (2015). Improving transitional patient safety: research protocol of the transitional incident prevention Programme. Safety. in Health.

[20] Farmacotherapeutisch compas. Zorginstituut Nederland. Available from: https://www.farmacotherapeutischkompas.nl

[21] Dutch guideline on medical correspondence between medical specialist and primary care provider. (in Dutch). [Richtlijn Informatie-uitwisseling tussen HuisArts en SPecialist bij verwijzingen (HASP)]. Utrecht: NHG. 2008. (new version becoming available in 2017). https://www.nhg.org/sites/default/files/content/nhg_org/uploads/richtlijn_hasp_0.pdf

[22] Dutch guideline on medication transfer, version 2008 (in Dutch). [Richtlijn Overdracht van medicatiegegevens in de keten, versie 1.0 d.d. 25 april 2008.] https://www.medicatieoverdracht.nl/Media/Default/richtlijn/Richtlijn_Overdracht_van_Medicatiegegevens-def-20080425.pdf

[23] Gandhi TK, Sittig DF, Franklin M, Sussman AJ, Fairchild DG, Bates DW (2000). Communication breakdown in the outpatient referral process. J Gen Intern Med.

[24] Dunnion ME, Kelly B (2005). From the emergency department to home. J Clin Nurs.

[25] Bell CM, Schnipper JL, Auerbach AD, Kaboli PJ, Wetterneck TB, Gonzales DV (2009). Association of communication between hospital-based physicians and primary care providers with patient outcomes. J Gen Intern Med.

[26] Kripalani S, LeFevre F, Phillips CO, Williams MV, Basaviah P, Baker DW (2007). Deficits in communication and information transfer between hospital-based and primary care physicians: implications for patient safety and continuity of care. JAMA.

[27] Sen S, Bowen JF, Ganetsky VS, Hadley D, Melody K, Otsuka S (2014). Pharmacists implementing transitions of care in inpatient, ambulatory and community practice settings. Pharm Pract (Granada).

[28] Hume AL, Kirwin J, Bieber HL, Couchenour RL, Hall DL, Kennedy AK (2012). Improving care transitions: current practice and future opportunities for pharmacists. Pharmacotherapy.

[29] Kattel S, Manning DM, Erwin PJ, Wood H, Kashiwagi DT, Murad MH. Information transfer at hospital discharge: a systematic review. J Patient Saf. 2016;00:00–0. 10.1097/PTS.0000000000000248 26741789

[30] Nemeth C, Wears R, Woods D, Hollnagel E, Cook R. Minding the Gaps: Creating Resilience in Health Care. In: Henriksen K, Battles JB, Keyes MA, et al., editors. Advances in Patient Safety: New Directions and Alternative Approaches (Vol. 3: Performance and Tools). Rockville (MD): Agency for Healthcare Research and Quality (US); 2008. 21249930

[31] Reason J (2000). Human error: models and management. BMJ.

